# Loss Aversion and Health Behaviors: Results from Two Incentivized Economic Experiments

**DOI:** 10.3390/healthcare9081040

**Published:** 2021-08-13

**Authors:** Donata Bessey

**Affiliations:** East Asia International College, Yonsei University, Wonju 26493, Korea; dbessey@yonsei.ac.kr; Tel.: +82-33-760-2276

**Keywords:** health behaviors, loss aversion, Big Five, personality traits, college students

## Abstract

Experimental research in health economics has analyzed the effects of economic preference parameters such as risk attitude and time preference on the probability of adopting risky health behaviors. However, the existing evidence is mixed and previous research often fails to include controls for other determinants of health behaviors such as personality traits. The aim of this research is to analyze the relationships between an incentivized measure of loss aversion and three health behaviors: smoking, binge drinking, and engaging in physical activity. Loss aversion is a preference measure that has been derived from prospect theory as an alternative approach to analyze decision-making under risk, such as the decision to invest in health capital, and has never been used in an analysis of the determinants of health behaviors before. Using two experimental samples of college students in the Republic of Korea and the United States of America, and controlling for Big Five personality traits and a host of individual-level control variables, there are no statistically significant relationships between loss aversion and the three aforementioned health behaviors, but relationships for Big Five conscientiousness, extraversion, agreeableness, and neuroticism. A candidate explanation might be lack of domain independence for loss aversion. Differences between the Korean and the US samples indicate the possibility of intercultural differences.

## 1. Introduction

There is a substantial literature of economic experiments using risk attitude and time preference to predict health behaviors, but the results are mixed (see [1] or [2] for surveys or [3]). Loss aversion is another candidate preference parameter to explain health behaviors and has been used in the design of behavioral programs to achieve health goals ([4,5] for smoking cessation or [6,7] for weight loss programs) in the context of health economic evaluations [8,9,10] or health insurance choice behaviors [11]. However, to the best of my knowledge, loss aversion has never been analyzed as a possible determinant of individual risky health behaviors before. Loss aversion refers to the prediction in prospect theory that individuals are typically more sensitive towards losses than towards gains [12], leading to the hypothesis that, with human capital being a risky investment ([13,14,15] for educational investment, Asano and Shibata’s [16] extension of Grossman’s [17] model for health investment), individuals who exhibit a higher degree of loss aversion should be less likely to engage in health behaviors linked to increased probabilities of fatal outcomes such as smoking. Those predictions from behavioral economic theory could be used in the design of prevention programs, especially when deciding about criteria for inclusion in programs. The following Table 1 briefly summarizes the concepts used in this research for readers with a non-economics background.

The aim of this research is to focus on three health behaviors that are linked to substantial negative health outcomes (consumption of alcohol, smoking, and (lack of) physical activity) and analyze their relationship with loss aversion. Mokdad et al. [18] find that tobacco, poor diet and physical inactivity, and alcohol consumption are the three leading preventable causes of death in the United States, providing the rationale to analyze these three behaviors. This research presents results from two incentivized experiments, one in the Republic of Korea (henceforth Korea) and one in the United States of America (henceforth US), adding to the growing body of literature linking economic lab preferences to field behavior [19].

In line with standard practice in experimental economics, undergraduate students were used as subjects, and previous research suggests that the college years are an important time period for developing health behaviors, providing the rationale to use a sample of college students. Undergraduate students often start developing behaviors such as eating habits causing weight gain [20,21], smoking, or drinking [22], and previous research suggests a strong link between early onset of smoking and drinking and dependency later in adulthood [23], as well as rising obesity levels in the US [24].

## 2. Materials and Methods

Data in Korea and in the US were collected in paper-and-pencil and online incentivized experiments, respectively, with a sample size of *n* = 170 for the Korean and *n* = 176 for the US data set. All subjects gave their informed consent for inclusion orally (in Korea) and by clicking on a “consent” button in the US before they participated in the study. The study was conducted in accordance with the Declaration of Helsinki, and the protocol was approved by the Institutional Review Board of Yonsei University, Mirae Campus (2018-52-0092).

Data in the US were collected in an online experiment. Subjects were undergraduate students in two and four-year colleges in California who were recruited online and on campuses. The online experiment was carried out using the eQuestionnaire platform. In the online experiment, all subjects had to go through instructions for the choice questions, a description of how payments would be carried out, and how the relevant decisions for payments would be determined. Participants received a show-up fee of $10 since the question aimed at eliciting loss aversion. Subjects were also informed that they would receive the payments immediately after their experimental session by certified mail, in the form of a check. 

Data in Korea were collected in a paper-and-pencil experiment due to the lack of an experimental laboratory at the university where the sessions took place. Subjects were freshmen students on a college campus in the Republic of Korea who were recruited using posters on campus and social network services. There were no restrictions based on major, age, or gender. The experimental instructions and questionnaires were translated into Korean by one person and back into English by a different person to check for inconsistencies. The instructions were pre-tested and the overall time needed for completion of all tasks and questionnaires was determined. Three experimental sessions were conducted later. In each session, subjects were first welcomed to the experiment, informed about the procedures, and provided their informed consent orally. Participants received a show-up fee of KRW 10,000 due to the question aimed at eliciting loss aversion. At the end of each experimental session, payments were determined and subjects were paid according to their choices in experimental tasks. For each of the tasks, one row was randomly determined by the throw of a die to be relevant for the payout. Payment was determined by the flip of a coin and corresponded to the choice that the individual made in the respective treatment. Due to university regulations forbidding cash payments, subjects received their payments in the form of a bank transfer immediately after the experimental sessions. On average, it took a participant 47 min to participate in the experiment and survey in the US, and the average payment was $39. In Korea, experimental sessions lasted on average 62 min, and the average payment was KRW 42,000. Since payments for both experiments were substantially above the two relevant minimum wages, there should be sufficient incentives for participation for the subjects. 

In both experiments, subjects answered a questionnaire on their health behaviors and individual background, a 15-item short version of the Big Five inventory [25,26], and a choice question in order to elicit loss aversion. 

The dependent variables (health outcomes of interest) were operationalized as follows: for smoking status, subjects were labeled as smokers if they answered that they smoke “daily” or “occasionally” as opposed to “not at all”. For reporting a binge drinking episode in Korea, subjects were labeled as having had a binge drinking episode if they answered that they had an episode “once”, “more than once” or “daily”, as opposed to those who answered “never”. In the US, the same question was used, but the numbers of drinks were different [eight for males/six for females]. For physical activity in Korea, those who reported any amount of physical activity were labeled as physically active. For physical activity in the US, those who reported physical activity as a hobby were labeled as physically active. These variables measuring physical activity were constructed in order to have a comparable measure in both samples, but cannot capture any compliance with guidelines regarding physical activity, such as the WHO recommendations [27].

In order to control for subjects’ personality, information on Big Five personality structure was included. The Big Five are five dimensions which define human personality at the broadest level [28,29]: openness to experience, conscientiousness, extraversion, agreeableness, and emotional stability or neuroticism. Health behavior models predict that personality traits are correlated with health habits, such as the decision to smoke [30]. Higher scores on the openness to experience and conscientiousness scale might be associated with resilience [30] and resulting differing choices to adopt behaviors, or differing responses to diagnoses. Higher scores on the neuroticism scale are associated with differences in experiencing negative emotions [30], such as stress, which might affect health behaviors and outcomes through various transmission channels. The Big Five have been shown to be associated with a number of health measures [30], and the associations seem to be strongest for conscientiousness and neuroticism [31,32]. Weston et al. [33] also find relationships between all Big Five personality traits except openness and individual disease, such as hypertension or diabetes, providing ample reasons to include information on Big Five personality traits in an analysis of determinants of health behaviors. For measurement, a 15-item short version of the Big Five inventory that was developed and validated for use in the German Socio-Economic Panel [25] was used and aggregated identically to the original research.

In order to control for family background, subjects’ answer to the question “how hard is it to raise $100 (KRW 100,000) for personal consumption for you?” was used as an income measure, with answer options ranging from 1 = very easy to 5 = very hard. Also, subjects’ number of siblings was included as an additional control variable for family background in order to control for possible differences in levels of social capital [34]. 

The second part of the experiments consisted of choice questions in order to elicit subjects’ degree of loss aversion using incentivized choices. Incentivized measures of economic preference parameters should be preferred for use in empirical research since subjects’ choices might differ from choices with hypothetical payoffs [35] and since subjects’ self-reported attitudes might be different from their actual choices [36]. Also, Croson et al. [37] argue that financial incentives may serve as a motivation device, with participants paying more attention to their choices and less noisy answers as a result. 

In both experiments, a measure of loss aversion developed by Fehr and Goette [38] in the adaptation of Gächter et al. [39] was used. In this choice task, subjects decided whether to accept or not six lotteries over gains and losses. In each lottery, the gain outcome was identical (KRW 6000 for the experiment in Korea, $6 for the experiment in the US) and the loss outcome increased between $2 (KRW 2000) and $7 (KRW 7000), with a 50% probability of each outcome. If subjects rejected a lottery, their payment was zero. At the end of the experiment, only one lottery was selected randomly for pay [40]. The decision sheet can be found in Appendix B. As a measure of loss aversion in regressions, the number of lotteries that the subject declined was used, with higher values corresponding to higher degrees of loss aversion. All estimations were run with the following set of control variables: Big Five personality traits, gender, age, an income measure, and the subjects’ number of siblings.

Regression analyses were carried out in STATA 16.1. Probit regressions were run and marginal effects were calculated by computing changes for each observation at its observed values and then averaged, using the mchange package [41]. 

## 3. Results

The three health behaviors analyzed here are binge drinking, smoking, and engaging in physical activity. In the two experimental samples analyzed in this research note, 11.18% of subjects in Korea are smokers (25.00% in the US), 69.41% reported having a binge drinking episode in Korea (30.68% in the US), and 77.06% reported engaging in physical activity (84.66% in the US). Complete summary statistics can be found in the following Table 2 and Table 3, and correlation matrices for both data sets can be found in Appendix A (Table A1 and Table A2). 

The following Table 4 and Table 5 present marginal effects from probit regressions for the following three outcomes: the probability of reporting a binge drinking episode, of being a smoker, and of engaging in physical activity, for the Korean and the US sample. ***, **, and * denote significance levels of 1%, 5%, and 10%, respectively.

Regression results from both experimental samples suggest that, when controlling for Big Five personality traits, gender, age, and two measures of family background, an experimental measure of loss aversion suggested by Gächter et al. [39] is not statistically significantly related to three health behaviors (namely, smoking, binge drinking, and physical activity). Regressions excluding the personality traits measures and using only the loss aversion measure suggest that this finding is not due to correlations between personality structure (i.e., all personality traits used in the regressions) and loss aversion (see Table A3 and Table A4 in Appendix A), as the finding persists in those regressions.

The Big Five personality traits show several correlations with the health behaviors analyzed. While there are no correlations for Big Five openness, Big Five conscientiousness is statistically significantly negatively correlated with the probability of reporting a binge drinking episode in the US sample, where an increase in one standard deviation of the conscientiousness scale is associated with a 7.0% lower probability of reporting a binge drinking episode.

Big Five extraversion is positively correlated with the probability of reporting a binge drinking episode, smoking, and engaging in physical activity in the Korean sample, and with the probability of reporting a binge drinking episode in the US sample. An increase in one standard deviation of the extraversion scale is associated with a 6.8% higher probability of reporting a binge drinking episode, a 7.1% higher probability of smoking, but also with a 7.1% higher probability of engaging in physical activity in the Korean sample, and with a 8.0% higher probability of reporting a binge drinking episode in the US sample.

Big Five agreeableness is positively correlated with engaging in physical activity in the Korean sample, where an increase of one standard deviation in the agreeableness scale is associated with a 6.8% increase in the probability of engaging in physical activity, and negatively correlated with the probability of reporting a binge drinking episode in the US sample, where an increase of one standard deviation of the agreeableness scale is associated with a 10.4% lower probability of reporting a binge drinking episode. Lastly, Big Five neuroticism is not correlated with any of the outcomes of interest in the Korean sample, but negatively correlated with the probability of reporting a binge drinking episode in the US sample. An increase of one standard deviation in the neuroticism scale is associated with a 10.8% decrease in the probability of reporting a binge drinking episode. 

For the remaining control variables, there are gender differences in the Korean sample, where being female is associated with a 9.8% lower probability of smoking, but also with a 26.2% lower probability of engaging in physical activity, with no differences found in the US sample. No age effects are found in the Korean sample, while in the US, older students are more likely to report a binge drinking episode and more likely to smoke. No statistically significant effects are found for the control variables aimed at capturing possible effects of family background, namely, number of siblings and a self-reported income measure.

## 4. Discussion

The aim of this research note was to test if loss aversion is a determinant of three risky health behaviors. The hypothesis that loss aversion should be negatively correlated with smoking and binge drinking and positively correlated with engaging in physical activity is rejected.

The first possible reason for this finding might be a lack of domain independence that has been cited for other preference parameters (Chapman and Elstein [42] and Lazaro [43] for time preference, Nicholson et al. [44] for risk attitude). For loss aversion, a similar argument could be made, and as a result, individuals’ behavior in incentivized choice experiments and when adopting health behaviors might differ. The second possible reason is purely due to the mechanics of statistical testing: lack of statistical power due to the relatively small sample sizes. Since this is, to the best of my knowledge, the first research to analyze the possible predictive power of loss aversion for health behaviors, there is no previous literature to compare this outcome to.

For the Big Five personality traits, this research note confirms some previous findings. While some earlier studies in health psychology suggested that correlations seemed to be strongest for conscientiousness and neuroticism [31,32], Weston et al. [33] found relationships between all traits except openness with individual diseases, such as hypertension or diabetes. This research also found relationships between three risky health behaviors and all Big Five personality traits except openness, partly confirming those previous research findings. 

The different age effects between the Korean and the US sample are likely due to differences in the minimum legal drinking and smoking ages in the two countries, with the minimum age being 18 in the Republic of Korea and 21 in the United States. The findings from this study underline possible changes in the application of theoretical concepts and empirical findings from behavioral economics to policy design in the field of public health. While loss aversion has been used in the design of programs to achieve weight loss and smoking cessation [4,5,6,7], its ability to predict the adoption of (un)healthy behaviors should be tested again using larger samples, if possible, drawn from the general population. In addition, the testing of the predictive power of existing experimental measures and the development of new experimental measures of loss aversion while addressing the possible lack of domain independence might be promising avenues for future research on the topic. Tests of other candidate measures beyond risk and time preference that might be useful as simple predictors for the decision to adopt health behaviors might further the understanding of determinants of lifestyle choices. 

Lastly, differences between the Korean and the US sample indicate the possibility of intercultural differences that might be investigated in further research.

## Figures and Tables

**Table 1:** Glossary of behavioral economics concepts used in this research.

Concept	Explanation
Preference parameters	Measures of individuals’ preferences over risk or time
Prospect theory	A behavioral economics approach to model individuals’ preferences over gains and losses when facing lottery-type choices
Loss aversion	A key prediction in prospect theory, suggesting that the loss in utility from losing an amount of money is larger than the gain in utility from winning the same amount
Human capital	An individual’s stock of skills, knowledge, and good health status
Domain independence	A preference parameter’s ability to explain behaviors across various decision-making domains (e.g., financial risk and health risk)

**Table 2:** Summary statistics, Korean sample.

Variable	Mean	Std. Dev.	Min	Max
Smoker	0.1118	0.3160	0	1
Binger	0.6941	0.4621	0	1
Physical activity	0.7706	0.4217	0	1
Loss aversion measure	3.7941	1.9609	0	6
Big Five Openness	4.7843	1.1395	1.6667	7
Big Five Conscientiousness	4.1745	0.9852	2	7
Big Five Extraversion	4.7216	1.2020	1	7
Big Fice Agreeableness	4.8549	0.9593	2.6667	6.6667
Big Five Neuroticism	4.6235	1.0809	1	7
Female	0.6118	0.4888	0	1
Age	19.2824	0.8301	18	24
Income measure	3.5176	0.9435	1	5
Number of siblings	1.1882	0.6342	0	4

**Table 3:** Summary statistics, US sample.

Variable	Mean	Std. Dev.	Min	Max
Smoker	0.2500	0.4342	0	1
Binger	0.3068	0. 4625	0	1
Physical activity	0.8466	0.3614	0	1
Loss aversion measure	3.3466	1.9969	0	6
Big Five Openness	5.8788	1.1175	1	7
Big Five Conscientiousness	5.4621	1.1046	2.3333	7
Big Five Extraversion	4.8920	1.1884	2	7
Big Fice Agreeableness	5.6345	1.1796	1	7
Big Five Neuroticism	3.6686	1.4768	1	7
Female	0.5114	0.5013	0	1
Age	21.5227	4.1747	17	31
Income measure	3.5568	1.2816	1	5
Number of siblings	1.2784	1.5664	0	9

**Table 4:** Korean sample, probit regressions, marginal effects.

Marginal Effects	Binge	Smoke	Physical Activity
Loss aversion	−0.002	0.002	−0.014
(+1)	[0.905]	[0.873]	[0.382]
Big Five openness	−0.013	0.014	−0.029
(+1 SD)	[0.737]	[0.605]	[0.401]
Big Five conscientiousness	−0.049	−0.005	−0.014
(+1 SD)	[0.230]	[0.835]	[0.694]
Big Five extraversion	0.068 **	0.071 **	0.071 **
(+1 SD)	[0.042]	[0.045]	[0.011]
Big Five agreeableness	0.025	0.011	0.068 **
(+1 SD)	[0.481]	[0.668]	[0.012]
Big Five neuroticism	0.024	0.016	0.012
(+1 SD)	[0.498]	[0.548]	[0.717]
Female	0.038	−0.098 ***	−0.262 ***
(+1)	[0.596]	[0.000]	[0.003]
Age	0.023	0.001	−0.038
(+1)	[0.572]	[0.978]	[0.310]
Income measure	0.031	0.026	−0.043
(+1)	[0.407]	[0.388]	[0.253]
Number of siblings	0.007	−0.040	−0.058
(+1)	[0.896]	[0.189]	[0.250]

Notes: This table presents average marginal effects that were calculated by computing changes for each observation at its observed values and then averaged, using the mchange package in STATA 16.1 [32]. These average marginal effects were calculated for an increase in one of the loss aversion measures. *p*-values are reported in brackets. The changes reported are for an increase in 1 for the loss aversion measure and for an increase in 1 standard deviation for the Big Five personality traits. ***, ** denote significance levels of 1%, 5%, respectively.

**Table 5:** US sample, probit regressions, marginal effects.

Marginal Effects	Binge	Smoke	Physical Activity
Loss aversion	−0.017	−0.013	0.009
(+1)	[0.284]	[0.394]	[0.483]
Big Five openness	−0.036	−0.018	−0.030
(+1 SD)	[0.293]	[0.585]	[0.424]
Big Five conscientiousness	−0.070 **	−0.029	0.008
(+1 SD)	[0.034]	[0.429]	[0.811]
Big Five extraversion	0.080 **	0.036	0.016
(+1 SD)	[0.028]	[0.322]	[0.535]
Big Five agreeableness	−0.104 ***	−0.029	−0.009
(+1 SD)	[0.001]	[0.396]	[0.799]
Big Five neuroticism	−0.108 ***	−0.019	0.009
(+1 SD)	[0.000]	[0.574]	[0.766]
Female	0.051	0.062	−0.089
(+1)	[0.135]	[0.383]	[0.224]
Age	0.015 **	0.024 ***	−0.004
(+1)	[0.044]	[0.001]	[0.502]
Income measure	−0.018	0.044	−0.003
(+1)	[0.446]	[0.113]	[0.879]
Number of siblings	0.021	0.011	0.001
(+1)	[0.305]	[0.593]	[0.939]

Notes: This table presents average marginal effects that were calculated by computing changes for each observation at its observed values and then averaged, using the mchange package in STATA 16.1 [32]. These average marginal effects were calculated for an increase in one of the loss aversion measures. *p*-values are reported in brackets. The changes reported are for an increase in 1 for the loss aversion measure and for an increase in 1 standard deviation for the Big Five personality traits. ***, ** denote significance levels of 1%, 5%, respectively.

## Data Availability

The data presented in this study are available on request from the author. The data are not publicly available due to data and privacy protection requirements.

## Appendix A. Summary Statistics and Correlation Matrices

**Table A1:** Correlation matrix, Korean sample.

	Smoker	Binger	Phys. Act.	Loss Aversion	Big 5 O.	Big 5 C.	Big 5 E.	Big 5 A.	Big 5 N.	Female	Age	Income	Siblings
Smoker	1												
Binger	0.0734	1											
Physical activity	0.1935	0.0021	1										
Loss aversion m.	0.0183	0.0085	−0.0217	1									
Big Five Openness	0.04	−0.0062	0.0113	−0.0862	1								
Big Five Conscient.	0.013	−0.077	0.0732	0.0687	0.0566	1							
Big Five Extraversion	0.0928	0.1298	0.0834	0.1086	0.1676	0.1867	1						
Big Five Agreeablen.	0.0603	0.0016	0.1513	0.0868	0.1973	0.3358	−0.0256	1					
Big Five Neuroticism	0.0142	0.0682	−0.0391	−0.0424	−0.1709	−0.2701	−0.1257	−0.131	1				
Female	−0.2537	0.0998	−0.205	0.0334	0.0223	−0.1288	0.2648	−0.1335	0.1025	1			
Age	0.0143	0.0259	−0.0336	0.0614	0.0919	0.1251	0.0911	0.1062	−0.149	−0.0782	1		
Income measure	0.0033	0.0667	−0.1162	−0.0092	−0.2001	−0.1678	−0.1817	−0.0603	0.229	0.1304	−0.1651	1	
Number of siblings	−0.0761	0.0159	−0.081	−0.0543	0.0456	0.0387	0.0614	−0.0067	0.0119	0.0081	0.1232	−0.0649	1

**Table A2:** Correlation matrix, US sample.

	Binger	Smoker	Phys. Act.	Loss Aversion	Big 5 O.	Big 5 C.	Big 5 A.	Big 5 E.	Big 5 N.	Age	Female	Income	Siblings
Binger	1												
Smoker	0.1849	1											
Physical activity	0.0439	0.0637	1										
Loss aversion m.	−0.0663	−0.0939	0.0424	1									
Big Five Openness	−0.0935	−0.0589	−0.0652	−0.0135	1								
Big Five Conscient.	−0.2046	−0.0913	−0.017	0.028	0.3887	1							
Big Five Agreeablen.	−0.2192	−0.0809	−0.034	0.0347	0.3077	0.5105	1						
Big Five Extraversion	0.1264	0.0452	0.0056	0.0086	0.2578	0.1765	0.2018	1					
Big Five Neuroticism	−0.104	0.0349	0.022	−0.0396	−0.2257	−0.389	−0.3641	−0.2538	1				
Age	0.1443	0.227	−0.0526	−0.0774	0.0088	0.0324	−0.0511	−0.0323	0.0304	1			
Female	0.0588	0.0656	−0.1007	0.1074	0.0739	−0.0439	0.0957	0.1124	0.063	−0.0138	1		
Income measure	−0.0874	0.0975	−0.0119	−0.0535	−0.0218	0.0473	0.0611	0.0309	0.1403	−0.106	0.1324	1	
Number of siblings	0.0549	0.0399	0.0052	−0.0712	−0.0143	−0.0759	0.0276	−0.0523	0.0179	−0.0136	−0.0077	0.0647	1

**Table A3:** Regression results with fewer control variables, Korean sample. ***, ** denote significance levels of 1%, 5%, respectively.

Regression resultss without personality traits and without any control variables, Korean sample, estimated coefficients.			
	Binge	Smoke	Phys. Act.
Loss aversion	0.0056	0.0160	−0.0153
	(0.0512)	(0.0664)	(0.0543)
Constant	0.4863 **	−1.2784 ***	0.7990 ***
	(0.2186)	(0.2856)	(0.2334)
**Regression results using only the loss aversion measure and control variables, Korean sample**			
	**Binge**	**Smoke**	**Phys. Act.**
Loss aversion measure	0.0036	0.0180	−0.0169
	(0.0518)	(0.0730)	(0.0558)
Female	0.2547	−0.9082 ***	−0.6166 **
	(0.2085)	(0.2815)	(0.2425)
Age	0.0699	0.0175	−0.0943
	(0.1261)	(0.1672)	(0.1234)
Income measure	0.0857	0.0579	−0.1588
	(0.1093)	(0.1439)	(0.1197)
Number of siblings	0.0315	−0.2911	−0.1617
	(0.1676)	(0.2498)	(0.1689)
Constant	−1.3429	−1.0647	3.8007
	(2.5138)	(3.3340)	(2.4905)

**Table A4:** Regression results with fewer control variables, US sample. ***, **, and * denote significance levels of 1%, 5%, and 10%, respectively.

Regression resultss without personality traits and without any control variables, US sample, estimated coefficients.			
	Binge	Smoke	Phys. Act.
Loss aversion measure	−0.0436	−0.0635	0.0330
	(0.0496)	(0.0513)	(0.0580)
Constant	−0.3609 *	−0.4672 **	0.9136 ***
	(0.1906)	(0.1947)	(0.2204)
**Regression results using only the loss aversion measure and control variables, US sample**			
	**Binge**	**Smoke**	**Phys. Act.**
Loss aversion measure	−0.0430	−0.0471	0.0400
	(0.0507)	(0.0537)	(0.0592)
Female	0.0414 *	0.0762 ***	−0.0174
	(0.0240)	(0.0251)	(0.0275)
Age	0.2081	0.1807	−0.3297
	(0.2044)	(0.2154)	(0.2374)
Income measure	−0.0961	0.1278	−0.0114
	(0.0801)	(0.0875)	(0.0942)
Number of siblings	0.0507	0.0347	0.0044
	(0.0644)	(0.0684)	(0.0748)
Constant	−1.0981	−2.7949 ***	1.4851 *
	(0.6714)	(0.7422)	(0.7853)

## Appendix B. Survey Questions and Scales

Experimental procedures.

All data in the US were collected in an online experiment. Subjects were undergraduate students in two- and four-year colleges in California who were recruited online and on campuses.

The online experiment was carried out using the eQuestionnaire platform. In the online experiment, all subjects had to go through instructions for the choice questions, a description of how payments would be carried out, and how the relevant decisions for payments would be determined. For the risk attitude questions, one row would be randomly selected for payment for every participant. For the time preference questions, every tenth participant would be picked for payment, and subjects were not told until after the experiment if they were chosen. In the intertemporal choice questions, the payment would again be determined by randomly selecting one row from the corresponding choice sheet, but only the picked subjects were actually paid. Subjects were also informed that they would receive the payments from the risk questions, as well as payments from the intertemporal choice questions if they preferred the immediate payment, immediately after the experimental session by certified mail, in the form of a check. Finally, they were also informed that all future payments would be sent by certified mail at the respective time in the future, in the form of a check. Participants received a show-up fee of $10 due to the question aimed at eliciting loss aversion.

All data in Korea were collected in a paper-and-pencil experiment. Subjects were freshmen students on a college campus in the Republic of Korea who were recruited using posters on campus and social network services. Data were collected in an economic paper-and-pencil experiment due to the lack of an experimental laboratory at the university where the sessions took place. In line with standard practice in experimental economics, undergraduate students were used as subjects, with no restrictions based on major, age, or gender, but the requirement that they must be freshmen. The experimental instructions and questionnaires were translated into Korean by one person and back into English by a different person to check for inconsistencies. The instructions were pre-tested and the overall time needed for completion of all tasks and questionnaires was determined. Three experimental sessions were conducted later. At the end of each experimental session, payments were determined and subjects were paid according to their choices in all experimental tasks. For each of the tasks, one row was randomly determined by the throw of a die to be relevant for the payout. Payment was determined identically and corresponded to the choice that the individual made in the respective treatment. In line with standard procedures in experimental economics, subjects were presented with incentivized choices. Participants received a show-up fee of $KRW 10,000 due to the question aimed at eliciting loss aversion.

**The following questions were used to construct the dependent variables of interest.**

**Smoking/nicotine consumption.**

At the present time, do you smoke cigarettes (including e-cigarettes) every day, occasionally or not at all?

1. Daily.
2. Occasionally.
3. Not at all.

**Binge drinking**

Now, some questions about your alcohol consumption. A ‘drink’ refers to:

- a bottle or small can of beer, cider or cooler with 5% alcohol content, or a small draft;
- a glass of wine with 12% alcohol content;
- a glass or cocktail containing 1 oz. of a spirit with 40% alcohol content.

How often in the last week have you had [5 for males/4 for females] or more drinks on one occasion?

1. Never.
2. Once.
3. More than once.
4. Every day.

**Physical activity**

In the last 7 days, on which days did you do these recreational activities that made you sweat at least a little and breathe harder? Please only include activities that lasted a minimum of 10 continuous minutes.

1. Monday: minutes.
2. Tuesday: minutes.
3. Wednesday: minutes.
4. Thursday: minutes.
5. Friday: minutes.
6. Saturday: minutes.
7. Sunday: minutes.

**The following task was used to elicit loss aversion (following [37]:**

In the following task, you have to decide if you want to choose a coin toss or not. If you choose the coin toss, you can win or lose money. If you choose the coin toss and lose money, you have to use the thank-you payment of 10,000 won (US$ 10) to cover your losses. If you do not choose the coin toss, nothing happens: you neither win nor lose money.

If you choose the coin toss, we will throw a coin at the end of today’s experiment, and your wins or losses will be as follows:

- If the coin comes up heads, you will win 6000 won (US$6).
- If the coin comes up tails, you will lose X won (US$X).

The amount of money that you will lose if the coin comes up tails differs between the different rows. In the table below, please decide for each row if you choose the coin toss or not. Again, if you choose the coin toss and the coin comes up heads, you will win 6000 won, but if the coin comes up tails, the corresponding amount you lose will be deducted from your gains at the end of the experiment.

At the end of the experiment, one of your choices will be randomly selected as the decision-that-counts for your payments, by rolling a die. As all choices are equally likely to be selected as the decision-that-counts, please think carefully if you choose the coin toss or not. According to your choice in the decision that will be selected as the decision-that-counts, we will either toss a coin that will determine your gains or losses, or nothing will happen.

	**I decline the coin toss** **(nothing will happen)**	**I choose the coin toss**
Tails: You lose 2000 won Heads: You win 6000 won	□	□
Tails: You lose 3000 won Heads: You win 6000 won	□	□
Tails: You lose 4000 won Heads: You win 6000 won	□	□
Tails: You lose 5000 won Heads: You win 6000 won	□	□
Tails: You lose 6000 won Heads: You win 6000 won	□	□
Tails: You lose 7000 won Heads: You win 6000 won	□	□

**The following questions were used to gather information about socio-economic background.**

Please answer the following questions about yourself.
How difficult would it be for you to raise 100,000 Won for personal consumption?
Very difficult-Difficult-Neutral-Easy-Very easy
What is your gender? Female-Male-Prefer not to say
What is your year of birth?
Do you have siblings? If yes, how many?

**The following scale was used for measurement of Big Five personality traits.**

In the next section, we list several characteristics that an individual might have. Most likely, you will completely agree that some characteristics describe you well, while others don’t, and you will be undecided on still others.

Again, please indicate the degree to which the following statements are true for you, on a scale from 1 (not like me at all) to 7 (very much like me).

I am a person who.

- Does a thorough job
- Is talkative
- Is sometimes rude to others
- Is original, comes up with new ideas
- Worries a lot
- Has a forgiving nature
- Tends to be lazy
- Is outgoing, sociable
- Values aesthetic, artistic experiences
- Gets nervous easily
- Does things efficiently
- Is reserved
- Is considerate and kind to almost everyone
- Has an active imagination
- Is relaxed, handles stress well
- Is eager to gain knowledge

## References

[1] Lawless L., Drichoutis A.C., Nayga R.M. (2013). Time Preferences and Health Behaviour: A Review. Agric. Food Econ..

[2] Story G.W., Vlaev I., Seymour B., Darzi A., Dolan R.J. (2014). Does Temporal Discounting Explain Unhealthy Behavior? A Systematic Review and Reinforcement Learning Perspective. Front. Behav. Neurosci..

[3] Conell-Price L., Jamison J. (2015). Predicting Health Behaviors with Economic Preferences & Locus of Control. J. Behav. Exp. Econ..

[4] Giné X., Karlan D., Zinman J. (2010). Put Your Money Where Your Butt Is: A Commitment Contract for Smoking Cessation. Am. Econ. J. Appl. Econ..

[5] Halpern S.D., French B., Small D.S., Saulsgiver K., Harhay M.O., Audrain-McGovern J., Loewenstein G., Brennan T.A., Asch D.A., Volpp K.G. (2015). Randomized Trial of Four Financial-Incentive Programs for Smoking Cessation. N. Engl. J. Med..

[6] Volpp K.G., John L.K., Troxel A.B., Norton L., Fassbender J., Loewenstein G. (2008). Financial Incentive-Based Approaches for Weight Loss: A Randomized Trial. JAMA J. Am. Med Assoc..

[7] John L.K., Loewenstein G., Troxel A.B., Norton L., Fassbender J.E., Volpp K.G. (2011). Financial Incentives for Extended Weight Loss: A Randomized, Controlled Trial. J. Gen. Intern. Med..

[8] Abellan-Perpiñan J.M., Bleichrodt H., Pinto-Prades J.L. (2009). The Predictive Validity of Prospect Theory versus Expected Utility in Health Utility Measurement. J. Health Econ..

[9] Attema A.E., Brouwer W.B.F., l’Haridon O. (2013). Prospect Theory in the Health Domain: A Quantitative Assessment. J. Health Econ..

[10] Attema A.E., Brouwer W.B.F., l’Haridon O., Pinto J.L. (2016). An Elicitation of Utility for Quality of Life under Prospect Theory. J. Health Econ..

[11] Kairies-Schwarz N., Kokot J., Vomhof M., Weßling J. (2017). Health Insurance Choice and Risk Preferences under Cumulative Prospect Theory—An Experiment. J. Econ. Behav. Organ..

[12] Kahneman D., Tversky A. (1979). Prospect Theory: An Analysis of Decision under Risk. Econometrica.

[13] Levhari D., Weiss Y. (1974). The Effect of Risk on the Investment in Human Capital. Am. Econ. Rev..

[14] Johnson W.R. (1978). A Theory of Job Shopping. Q. J. Econ..

[15] Gibbons R., Murphy K.J. (1992). Optimal Incentive Contracts in the Presence of Career Concerns: Theory and Evidence. J. Political Econ..

[16] Asano T., Shibata A. (2011). Risk and Uncertainty in Health Investment. Eur. J. Health Econ..

[17] Grossman M. (1972). On the Concept of Health Capital and the Demand for Health. J. Political Econ..

[18] Mokdad A.H., Marks J.S., Stroup D.F., Gerberding J.L. (2004). Actual Causes of Death in the United States, 2000. J. Am. Med Assoc..

[19] Barberis N.C. (2013). Thirty Years of Prospect Theory in Economics: A Review and Assessment. J. Econ. Perspect..

[20] Lloyd-Richardson E.E., Bailey S., Fava J.L., Wing R. (2009). A Prospective Study of Weight Gain during the College Freshman and Sophomore Years. Prev. Med..

[21] Racette S.B., Deusinger S.S., Strube M.J., Highstein G.R., Deusinger R.H. (2005). Weight Changes, Exercise, and Dietary Patterns during Freshman and Sophomore Years of College. J. Am. Coll. Health.

[22] Grace T.W. (1997). Health Problems of College Students. J. Am. Coll. Health.

[23] Chen K., Kandel D.B. (1995). The Natural History of Drug Use from Adolescence to the Mid-Thirties in a General Population Sample. Am. J. Public Health.

[24] Fryar C.D., Carroll M.D., Ogden C.L. (2012). Prevalence of Overweight, Obesity, and Extreme Obesity among Adults: United States, Trends 1960–1962 through 2009–2010.

[25] Gerlitz J.-Y., Schupp J. (2005). Zur Erhebung Der Big-Five-Basierten Persönlichkeitsmerkmale Im SOEP. Res. Notes.

[26] Lang F.R., John D., Lüdtke O., Schupp J., Wagner G.G. (2011). Short Assessment of the Big Five: Robust across Survey Methods except Telephone Interviewing. Behav. Res. Methods.

[27] Bull F., Al-Ansari S., Biddle S., Borodulin K., Buman M.P., Cardon G., Carty C., Chaput J.P., Chastin S., Chou R. (2020). World Health Organization 2020 guidelines on physical activity and sedentary behaviour. Br. J. Sports Med..

[28] Goldberg L.R., McReynolds P. (1971). A historical survey of personality scales and inventories. Advances in Psychological Assessment.

[29] Goldberg L.R. (1993). The Structure of Phenotypic Personality Traits. Am. Psychol..

[30] Smith T.W. (2006). Personality as Risk and Resilience in Physical Health. Curr. Dir. Psychol. Sci..

[31] Turiano N.A., Pitzer L., Armour C., Karlamangla A., Ryff C.D., Mroczek D.K. (2012). Personality Trait Level and Change as Predictors of Health Outcomes: Findings from a National Study of Americans (MIDUS). J. Gerontol. Ser. B Psychol. Sci. Soc. Sci..

[32] Friedman H.S., Kern M.L. (2014). Personality, Well-Being, and Health. Annu. Rev. Psychol..

[33] Weston S.J., Hill P.L., Jackson J.J. (2015). Personality Traits Predict the Onset of Disease. Soc. Psychol. Personal. Sci..

[34] Bourdieu P., Richardson J. (1986). The forms of capital. Handbook of Theory and Research for the Sociology of Education.

[35] Anderson L.R., Mellor J.M. (2008). Predicting Health Behaviors with an Experimental Measure of Risk Preference. J. Health Econ..

[36] Glaeser E.L., Laibson D.I., Scheinkman J.A., Soutter C.L. (2000). Measuring Trust. Q. J. Econ..

[37] Croson R., Schultz K., Siemsen E., Yeo M.L. (2013). Behavioral Operations: The State of the Field. J. Oper. Manag..

[38] Fehr E., Goette L. (2007). Do Workers Work More If Wages Are High? Evidence from a Randomized Field Experiment. Am. Econ. Rev..

[39] Gächter S., Johnson E., Herrmann A. (2007). Individual-Level Loss Aversion in Riskless and Risky Choices. http://ftp.iza.org/dp2961.pdf.

[40] Cubitt R.P., Starmer C., Sugden R. (1998). On the Validity of the Random Lottery Incentive System. Exp. Econ..

[41] Long J.S., Freese J. (2014). Regression Models for Categorical Dependent Variables Using Stata.

[42] Chapman G.B., Elstein A.S. (1995). Valuing the Future: Temporal Discounting of Health and Money. Med Decis. Mak..

[43] Lazaro A. (2002). Theoretical Arguments for the Discounting of Health Consequences: Where Do We Go from Here?. PharmacoEconomics.

[44] Nicholson N., Soane E., Fenton-O’Creevy M., Willman P. (2005). Personality and Domain-Specific Risk Taking. J. Risk Res..

